# Expression and prognosis analysis of mitochondrial ribosomal protein family in breast cancer

**DOI:** 10.1038/s41598-022-14724-7

**Published:** 2022-06-23

**Authors:** Xiaoyi Lin, Lijuan Guo, Xin Lin, Yulei Wang, Guochun Zhang

**Affiliations:** 1 Department of Breast Surgery, Guangdong Provincial People’s Hospital, Guangdong Academy of Medical Sciences, Guangzhou, Guangdong China; 2 Shantou University Medical College, Shantou, Guangdong China; 3 School of Medicine, South China University of Technology, Guangzhou, Guangdong China; 4 The Second School of Clinical Medicine, Southern Medical University, Guangzhou, Guangdong China

**Keywords:** Biochemistry, Biological techniques, Cancer

## Abstract

Breast cancer (BC) is characterized by high morbidity. Mitochondrial ribosomal protein (MRP) family participates in mitochondrial energy metabolism, underlying BC progression. This study aims to analyze the expression and prognosis effect of the MRP genes in BC patients. GEPIA2, UALCAN, cBioPortal, and MethSurv were used to demonstrate the differential expression, genomic alteration profiles, and DNA methylation of the MRP gene family in BC. Functional enrichment analysis and protein–protein interaction network construction were performed to understand the biological function. Based on 1056 TCGA samples with the transcriptional level of MRPs, Kaplan–Meier curves, Cox, and LASSO regression were applied to explore their prognostic effects. 12 MRPs were upregulated in BC, which were associated with gene amplification and DNA methylation. MRP genetic alteration occurred in 42% of BC patients, and amplification was the most frequent variation. Functioning in its entirety, the MRP family was involved in mitochondrial translational termination, elongation, translation, and poly(A) RNA binding. High expression of MRPL1, MRPL13, MRPS6, MRPS18C, and MRPS35, as well as low levels of MRPL16, and MRPL40 significantly indicated poor prognosis in BC patients. Thus, a novel MRP-based prognostic nomogram was established and verified with favorable discrimination and calibration. We not only provided a thorough expression and prognosis analysis of the MRP family in BC patients but also constructed an MRP-based prognostic nomogram. It was suggested that MRPs acted as biomarkers in individualized risk prediction and may serve as potential therapeutic targets in BC patients.

## Introduction

Breast cancer (BC) is the most commonly diagnosed female malignancy, with an increasing rate of about 0.5% annually^1^. As the second leading cause of death, many clinicopathological biomarkers have been utilized to predict, and therefore improve its survival^2–4^. However, BC is a highly heterogeneous cancer^5^, and conventional approaches such as TNM staging and molecular subtypes are insufficient. Fortunately, the recent development of molecular signatures extensively promoted the survival of BC patients and the development of targeted therapy^6–8^. It leads to the great importance of gene expression profiling in prognosis prediction and high-risk BC patient selection.

Genome instability fosters aberrant hallmark functions of cancer, one of which is mitochondrial energy metabolism and apoptosis^9,10^. Mitochondrial ribosomal proteins (MRPs), encoded by the nuclear MRP gene family^11^, are indispensable components in mitochondrial translation. In previous studies, more than 40 MRPs were reported to be overexpressed in BC, acting as promotors of BC cellular viability^12,13^. Recently, X Li et al. reported MRPL52 upregulation in BC and its oncogenic role in apoptotic resistance and metastatic promotion^14^. Some MRPs serve as tumor suppressors. For example, MRPL41 is downregulated in BC^15^. Its upregulation was associated with positive estrogen receptor status and could be induced by estradiol and trichostatin A^16^.

MRP family members are important regulators in BC development, but the prognostic values for BC patients have not been elucidated. In this study, we conducted a comprehensive bioinformatics analysis of the MRP family. The prognostic effect was investigated, and an MRP-based model was further established to predict the BC survival. We aimed to help clinicians to predict the risk of BC patients and provide researchers with new therapeutic candidates.

## Material and methods

### Differential expression analysis

Gene Expression Profiling Interactive Analysis (GEPIA2), a web server with RNA sequencing expression data^17^, was used to find out the differentially expressed genes (DEGs) in BC compared with normal tissue. Based on the analysis of variance (ANOVA), the Log2(Fold Change) cutoff was 1 and the *P* value cutoff was 0.001. 1085 BC samples were analyzed and matched with 291 samples from TCGA normal and GTEx data. The DEGs were screened for subsequent molecular analysis. Additionally, the differential expression of MRP genes in various primary tumors, as well as BC cell lines, were validated in the Cancer Cell Line Encyclopedia (CCLE) database^18^. Due to the high heterogeneity of BC, we further explored the DEGs in different BC subtypes relative to normal tissue using GEPIA2, with the same criteria and matched normal data mentioned above. Differential protein expression of BC was also explored based on the Clinical Proteomic Tumor Analysis Consortium (CPTAC) Confirmatory/Discovery dataset in UALCAN^19,20^.

### Genomic alteration and methylation analysis

cBioPortal is a platform containing multidimensional genomics datasets^21^, and was utilized to visualize the genomic alteration of DEGs and to evaluate the mRNA expression correlation with DNA copy number variation. Copy number alteration was determined using GISTIC 2.0, in which a value of 2 is equivalent to amplification. Besides genetic mutation and copy-number alteration, DNA methylation also plays a vital role in cancer progression^9^. Therefore, the Spearman correlations between MRP methylation and RNA expression (RNA Seq V2 RSEM, log2(value + 1)) were conducted. Moreover, we explored the prognostic values of genetic alteration and methylation levels in BC by cBioportal and MethSurv, respectively. MethSurv is an online tool providing survival analysis based on DNA methylation data from the TCGA database^22^. We employed a “single CpG” analysis module, and all the available genomic regions and individual CpG sites were evaluated. BC patients were dichotomized by median methylation levels, and the log-likelihood ratio (LR) test *P* value < 0.05 was considered significantly different in prognosis.

### Functional enrichment analysis and protein interaction visualization

Gene Ontology (GO) represents the biological function of candidate genes^23^, and the Database for Annotation, Visualization, and Integrated Discovery (DAVID) was applied to conduct GO enrichment analysis^24,25^. Since proteins form protein complexes and play biological roles synergistically, protein–protein interaction (PPI) network construction would be essential to understanding MRPs. String, a database integrating all known physical and functional protein interactions^26^, was employed to explore the MRP family members. Cytoscape was adopted to integrate and modify the network^27^. The above-mentioned Log2 (Fold Change) was used to represent the node size, indicating the significance of expression difference.

### Data acquisition

1056 Primary BC samples with complete transcriptome data and clinical traits in the TCGA database were acquired via the UCSC Xena platform^28^, after excluding cases with incomplete overall survival (OS) information, or unknown age and TNM staging. The TNM stage was decided by the American Joint Committee on Cancer Tumor Stage Code. The status of hormone receptor (HR) and human epidermal growth factor receptor 2 (HER2) were assessed by immunohistochemistry (IHC) or fluorescence in situ hybridization (FISH). The mRNA profiles were measured using the Illumina HiSeq 2000 RNA Sequencing platform and shown as log2(value + 1) transformed RSEM normalized count. 77 MRP family members were included in the gene expression profiles, except MRPL57 which was not covered in the TCGA dataset. Based on the median level of each MRP, the samples were classified into high- and low- expression groups.

### Survival analysis and prognostic model establishment

We performed univariate Cox proportional hazards regression to determine the prognostic factors for OS among all 77 MRP family members. The significant prognostic effects of MRPs were plotted using Kaplan–Meier curves. The least absolute shrinkage and selection operator (LASSO) regression was performed to exclude the collinearity between variables and improve the accuracy of the model. Stepwise multivariate Cox regressions were carried out to select the optimal combination of covariates. Based on the combined results, we developed an MRP-based prognostic signature, evaluated by calibration curves and the area under the curve (AUC) of the time-dependent receiver operating characteristic (ROC) curves.

### Statistical analysis

Statistical analysis was performed using R 4.0.2 software. All statistical tests were two-sided. A *P* value less than 0.05 was used as a cutoff for statistical significance.

### Ethical approval

There was no need for ethical approval as all data analyzed were obtained from public databases.

## Results

### Identification of differentially expressed MRP family members

**Figure 1:**
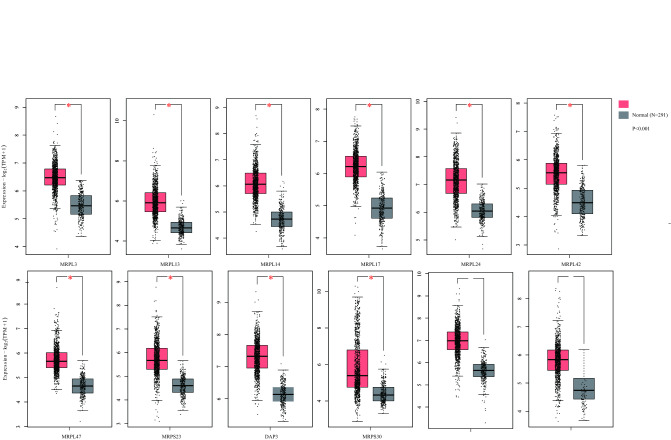
Differential expression of MRP genes between breast cancer and normal tissues (GEPIA2). Red and grey box plots shown the expression of breast tumor and normal tissues respectively. The asterisk (*) indicated significant differences (*P* < 0.001) between the two groups.

In the MRP family, 12 members were specifically upregulated in BC (*P* < 0.001) and thus identified as differentially expressed genes (DEGs), including MRPL3, MRPL13, MRPL14, MRPL17, MRPL24, MRPL42, MRPL47, MRPS23, DAP3, MRPS30, MRPS34, MRPS35 (Fig. 1). The most overexpressed gene was MRPL14, with a Log2 (Fold Change) of 1.336, followed by MRPS34, with a Log2 (Fold Change) of 1.333. Among various types of cancer, the DEGs expressions of BC were comparable to other primary tumors (Fig. S1). But the median expressions of MRPL3, MRPL13, MRPL14, MRPL24, MRPL47, DAP3, and MRPS34 were higher than the average. The mRNA levels of DEGs across different BC cell lines were then illustrated in Fig. S2. It was noteworthy that all DEGs’ expressions were the lowest on BC cell line SUM102PT. Low expressions of MRPL3, MRPL42, MRPL47, and low expressions of MRPL13, MRPL14, MRPS23 were detected in HDQP1 and SUM185PE cells, respectively. Moreover, we discovered that MRP genes were upregulated in a subtype-specific manner (Table 1). Half of the 12 DEGs (MRPL13, MRPL14, MRPL17, MRPL42, DAP3, and MRPS35) were highly upregulated in all the subtypes. Apart from the above-mentioned MRP genes, high expression of MRPS28 was specifically observed in the Luminal B subtype, MRPL27 in the HER2-enriched subtype, and MRPL9, MRPL37, MRPL41, MRPL48, MRPS12 in the basal-like subtype. These results suggested the oncogenic effects of MRP family genes on BC progression.

**Table 1:** Differential MRP gene expression in different breast cancer subtypes.

Subtypes	Differential expressed genes (Up-regulated)
Luminal A (n = 415)	MRPL13, MRPL14, MRPL17, MRPL24, MRPL42, MRPS23, DAP3, MRPS30, MRPS34, MRPS35
Luminal B (n = 192)	MRPL3, MRPL12, MRPL13, MRPL14, MRPL15, MRPL17, MRPL19, MRPL24, RPL30, MRPL35, MRPL42, MRPL47, MRPL51, MRPS16, MRPS23, MRPS28, DAP3, MRPS30, MRPS34, MRPS35
HER2-enriched (n = 65)	MRPL3, MRPL13, MRPL14, MRPL17, MRPL19, RPL27, MRPL30, MRPL35, MRPL42, MRPL47, MRPS16, MRPS23, DAP3, MRPS34, MRPS35
Basal-like (n = 135)	MRPL3, MRPL9, MRPL12, MRPL13, MRPL14, MRPL15, MRPL17, MRPL19, MRPL37, MRPL41, MRPL42, MRPL47, MRPL48, MRPL51, MRPS12, DAP3, MRPS35

### Genetic and epigenetic features, and the correlation with MRP expression

**Figure 2:**
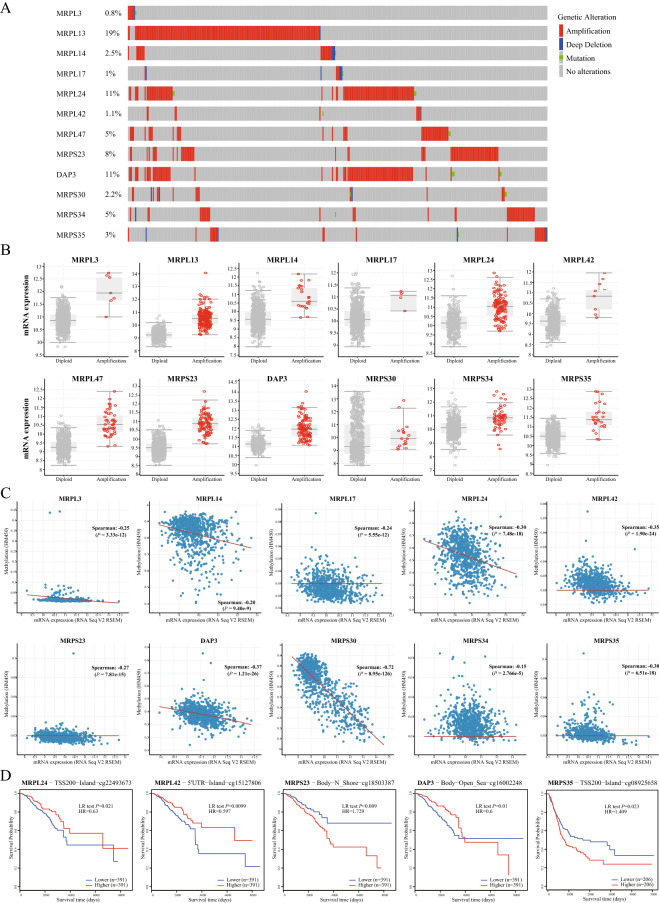
Gene alteration and methylation of MRP genes in breast cancer. (**A**) Summary of alteration patterns. The proportion of samples with genetic alteration was expressed as a percentage next to the gene, and genetic alteration types were indicated in bars with different colors. (**B**) The impact of gene amplification on the transcriptional level. Grey and red circles represented the mRNA expression in samples with diploid genotype and gene amplification. (**C**) Correlation between DNA methylation and mRNA expression in breast cancer. Each sample was indicated in a blue dot, and the fitted line was in red. The Spearman correlation coefficient and *P* value examined the association. (**D**) Kaplan–Meier plots showing overall survival in BC patients with hypermethylation (red curves) and hypomethylation (blue curves) for cg22493673-MRPL24, cg15127806-MRPL42, cg18503387-MRPS23, cg16002248-DAP3, and cg08925658-MRPS35. Log-likelihood ratio (LR) test *P* values were used to determine the prognostic significance and the hazard ratio (HR) was a relative prognostic measure of BC patients with hypermethylation compared with those with hypomethylation.

Using cBioPortal, genomic alteration analyses of DEGs in the MRP family were conducted. Overall, aberrant alterations occurred in 42% of the samples. As shown in Fig. 2A, MRPL13 (19%), MRPL24 (11%), and DAP3 (11%) were altered most frequently. Genetic amplification was the most common variation. A strong correlation was revealed between gene amplification and higher levels of the corresponding mRNA for most of the DEGs, such as MRPL13, MRPL14, and MRPS35 (Fig. 2B). As for epigenetic modification, all the DEGs’ expressions were negatively correlated with the methylation level of the MRP gene family in BC (Fig. 2C), except MRPL13 and MRPL47, whose methylation data was not included in cBioPortal. In addition, the genetic alterations of DEGs were not significantly associated with prognosis (Fig. S3), whereas the methylation levels of DEGs were remarkably correlated with OS (Fig. 2D). Hypermethylation of MRPL24 promoter, MRPL42 promotor, and CpG cg16002248 annotated to the gene DAP3 resulted in better OS in BC patients. By contrast, patients with high MRPS23 and MRPS35 methylation had worse OS.

### MRP protein expression and functional enrichment analysis

**Figure 3:**
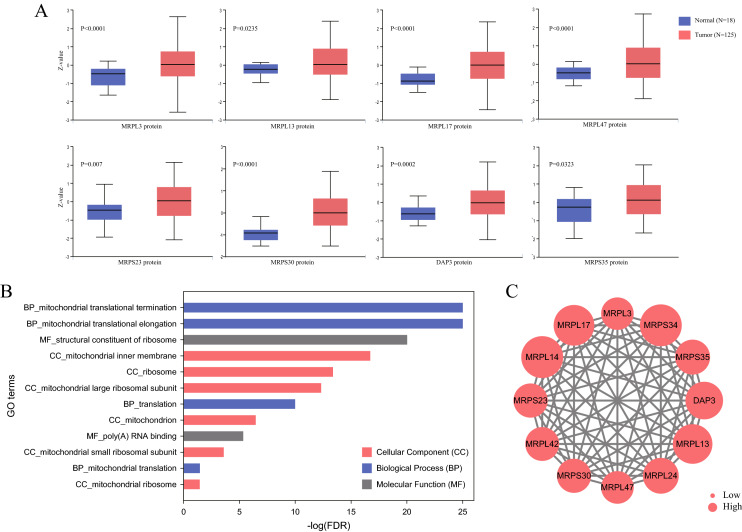
Proteomic and functional analysis of MRPs in breast cancer. (**A**) Differential proteomic levels of MRP genes between BC and normal tissues (CPTAC). (**B**) Gene Ontology enrichment analysis of MRP genes. (**C**) Protein–protein interaction network of MRP genes. The node size was proportional to Log2 (Fold Change).

At translational levels, among the 12 DEGs, MRPL3, MRPL13, MRPL17, MRPL47, MRPS23, MRPS30, DAP3, and MRPS35 exhibit significant differences in proteomic expression based on the CPTAC dataset. As demonstrated in Fig. 3A, the levels of these 8 proteins in BC were prominently higher than in normal tissues. To probe the biological function of MRPs, GO enrichment analysis of DEGs was performed. It was recognized that they were involved in mitochondrial translational termination, elongation, and translation, and were mainly located on the mitochondrial inner membrane, ribosome, and mitochondrial large ribosomal subunit (Fig. 3B). As for molecular function, they primarily played roles in the structural constituents of ribosomes and poly(A) RNA binding. The interplay among the DEGs was revealed using the PPI network (Fig. 3C). Remarkably, all of them interacted with each other, implying the MRP family functioned in its entirety.

### The prognostic value of MRP expression in breast cancer

**Figure 4:**
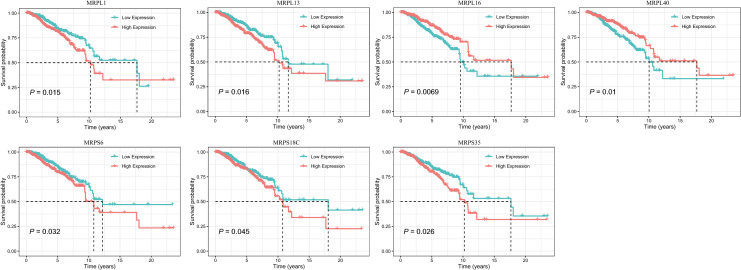
Kaplan–Meier survival curves based on differential MRP gene expression levels in BC patients. Low and high expression groups were represented in green and red respectively. Black dashed lines shown the median survival time.

Regarding BC prognosis, all 77 MRP genes were incorporated in univariate Cox regression (Table S1). As summarized in Table 2, the mRNA expression of 7 MRPs were revealed to be associated with OS in BC. Age (HR, 1.034; 95% CI, 1.021–1.047; *P* < 0.001), stage (III vs I: HR, 3.026; 95% CI, 1.706–5.368; *P* < 0.001; IV vs I: HR, 13.042; 95% CI, 6.430–26.453; *P* < 0.001) and IHC-based subtypes (HR-/HER2- vs HR + /HER2-,1.811; 95% CI, 1.125–2.914; *P* = 0.015) were also prognostic factors for BC. In Fig. 4, Kaplan–Meier survival curves were plotted to assess the effects of differentially expressed MRPs on OS. It was manifested that higher transcription levels of MRPL1 (HR, 1.518; 95% CI, 1.082–2.128; *P* = 0.016), MRPL13 (HR, 1.514; 95% CI, 1.079–2.123; *P* = 0.016), MRPS6 (HR, 1.440; 95% CI, 1.030–2.014; *P* = 0.033), MRPS18C (HR, 1.405; 95% CI, 1.005–1.963; *P* = 0.047), and MRPS35 (HR, 1.459; 95% CI, 1.044–2.039; *P* = 0.027) were correlated with deterioration of prognosis. Conversely, patients with downregulation of MRPL16 (HR, 0.630; 95% CI, 0.449–0.884; *P* = 0.007), and MRPL40 (HR, 0.643; 95% CI, 0.458–0.903; *P* = 0.011) had significantly shorter survival.

**Table 2:** Univariate Cox proportional hazards regression analysis in the TCGA patients, and multivariate analysis of the MRP-based nomogram.

Variables	Univariate analysis		Multivariate analysis	
	Hazard ratios (95% CI)	*P* value	Hazard ratios (95% CI)	*P* value
Age	1.034 (1.021–1.047)	< 0.001*	1.037 (1.023–1.050)	< 0.001*
**Stage**				
I	1	–	1	
II	1.574 (0.910–2.721)	0.104	1.754 (1.007–3.055)	0.047
III	3.026 (1.706–5.368)	< 0.001*	3.509 (1.952–6.307)	< 0.001*
IV	13.042 (6.430–26.453)	< 0.001*	15.058 (7.254–31.260)	< 0.001*
**Subtype**				
HR +/HER2 −	1		1	
HR +/HER2 +	1.380 (0.798–2.386)	0.249	1.201 (0.682–2.117)	0.525
HR −/HER2 +	2.119 (0.962–4.666)	0.062	2.454 (1.098–5.484)	0.029
HR −/HER2 −	1.811 (1.125–2.914)	0.015*	2.349 (1.421–3.885)	< 0.001*
Unknown	1.664 (1.063–2.606)	0.026*	1.483 (0.942–2.333)	0.089
**MRPL1 expression**				
Low	1	–	–	
High	1.518 (1.082–2.128)	0.016*	–	
**MRPL13 expression**				
Low	1	–	–	
High	1.514 (1.079–2.123)	0.016*	–	
**MRPL16 expression**				
Low	1	–	1	
High	0.630 (0.449–0.884)	0.007*	0.663 (0.456–0.963)	0.031*
**MRPL40 expression**				
Low	1	–	1	
High	0.643 (0.458–0.903)	0.011*	0.567 (0.391–0.822)	0.003*
**MRPS6 expression**				
Low	1	–	–	
High	1.440 (1.030–2.014)	0.033*	–	
**MRPS18C expression**				
Low	1	–	1	
High	1.405 (1.005–1.963)	0.047*	1.412 (0.991–2.012)	0.056
**MRPS35 expression**				
Low	1	–	1	
High	1.459 (1.044–2.039)	0.027*	1.480 (1.047–2.092)	0.027*

*HR* hormone receptor, *HER2* human epithelial growth factor receptor, *CI* confidence intervals.

*P value < 0.05, the variable has statistically significance.

### Development of an MRP-based prognostic model

**Figure 5:**
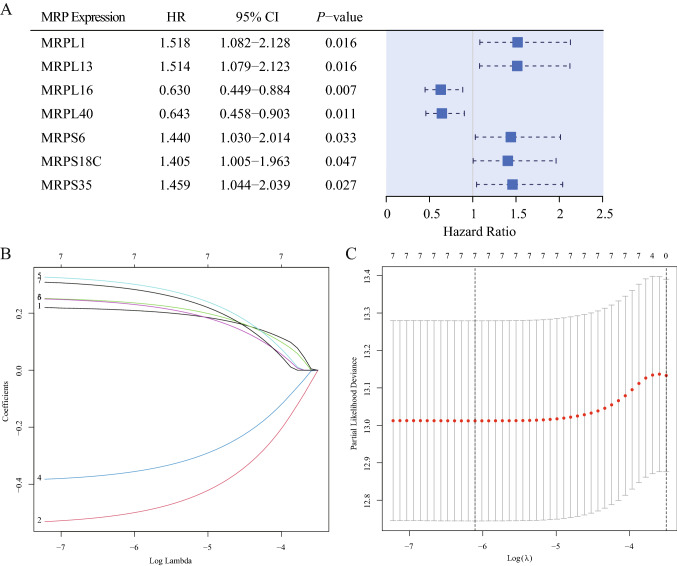
Construction of an MRP-based nomogram. (**A**) Forest plot of univariate Cox regression analysis of MRP genes. (**B**) Least absolute shrinkage and selection operator (LASSO) coefficient profiles of the 7 MRPs determined by the optimal lambda. (**C**) Selection result of the optimal lambda in the model. The partial likelihood deviance (binomial deviance) curve was drawn versus log(λ), and vertical dotted lines were made at the optimal values.

**Figure 6:**
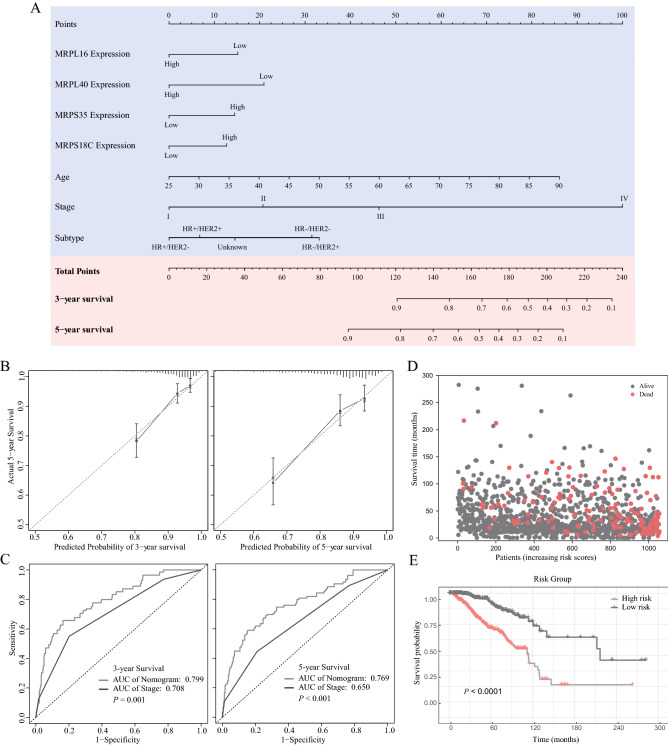
MRP-based prognostic model and its evaluation. (**A**) A nomogram to predict the overall survival in BC patients. (**B**) Calibration curves of the nomogram to predict the probability of 3-year and 5-year overall survival. (**C**) Time-dependent receiver operating characteristic curves for nomogram-based 3-year and 5-year overall survival prediction. (**D**) Patient distribution based on different risk scores. (**E**) Kaplan–Meier survival curves of different risk groups in BC patients.

The prognostic effects of 7 MRPs were displayed in a forest plot (Fig. 5A) using HR (Hazard Ratio) and 95% confidence intervals (CI). Given the striking effect of MRPs on the BC survival outcome, an MRP-based prognostic model was developed to predict the OS. Firstly, the 7 MRPs were subjected to LASSO regression. The model fit the best when the penalty coefficient was 7 (Fig. 5B, C). Therefore, all MRPs were selected for stepwise multivariate Cox regression analyses. After excluding the variates without significant statistical difference and comparing the concordance index, 4 MRPs (MRPL16, MRPL40, MRPS18C, and MRPS35), age, and stage were integrated into the nomogram. Table 2 displayed the multivariate Cox regression analysis of the markers included in the nomogram. Patients with higher transcription levels of MRPS18C (HR, 1.412; 95% CI, 0.991–2.012; *P* = 0.056) and MRPS35 (HR, 1.48; 95% CI, 1.047–2.092; *P* = 0.027) indicated significantly shorter OS, while upregulated MRPL16 (HR, 0.663; 95% CI, 0.456–0.963; *P* = 0.031) and MRPL40 (HR, 0.567; 95% CI, 0.391–0.822; *P* = 0.003) were identified as independent protective factors. The nomogram was demonstrated in Fig. 6A. According to the nomogram scoring system, each variable was given a nomogram score on the point scale. They were summed up and a total nomogram score was obtained for each patient, acquiring a predicted probability of OS. Based on the median value of total scores, TCGA patients were stratified into high-risk and low-risk groups.

### MRP-based prognostic nomogram evaluation

Calibration plots (Fig. 6B) confirmed good consistency between the prediction of the nomogram and actual observations. The 3-year and 5-year AUC values of the nomogram for predicting OS were 0.799 (95% CI, 0.737–0.860) and 0.769 (95% CI, 0.705–0.83.4) respectively, compared with 0.708 (95% CI, 0.643–0.773, *P* = 0.001), and 0.650 (95% CI, 0.585–0.715, *P* < 0.001) for cancer stage (Fig. 6C). Hence, the MRP-based nomogram had superior predictive accuracy in BC survival outcomes. The distribution diagram of risk scores and survival status (Fig. 6D), and Kaplan–Meier curves of different risk groups (Fig. 6E) suggested the survival differences between the high- and low-risk groups.

## Discussion

MRP gene family is implicated in biogenesis, metabolism, and apoptosis in BC cells. Defects in mitochondrial function underlying cancer growth may arise from abnormal gene profiling^9,29^. Simultaneously, they can modify gene expression and genomic stability^30^. To understand the aberrant MRP expression and alteration in BC, our research provided thorough bioinformatics analysis of the MRP gene family. According to the results, 12 MRPs (MRPL3, MRPL13, MRPL14, MRPL17, MRPL24, MRPL42, MRPL47, MRPS23, DAP3, MRPS30, MRPS34, MRPS35) were associated with the occurrence of BC. Consistent with previous research, higher expressions of MRPL13 and MRPS23 were revealed in BC tissues and associated with poor prognosis^31–34^. Interestingly, downregulation of MRPL13 restrained the proliferation and migration of BC^35^, whereas it enhanced the invasiveness of hepatoma via the reactive oxygen species (ROS)-Claudin-1 pathway^36^. We also discovered that MRP expression varied with different BC cell lines and subtypes. The lowest expressions of 12 MRP genes were observed on BC cell line SUM102PT. It was acquired from a minimally invasive human BC, characterized by overexpression of EGFR without gene amplification^37^. Basal-like BC, the most aggressive subtype, had the most up-regulated MRP genes. Upregulation of mRNA expression level was correlated with relevant gene amplification, the most frequent genetic alteration in MRPs, as well as DNA hypomethylation. L Liu et al. found that methylation of MRPS23 led to MRPS23 degradation and promote BC metastasis^38^. Our survival analysis further validated that hypermethylation of MRPS23 could result in poor survival outcomes.

In our study, the biological function of mitochondrial translational termination, elongation, and translation, as well as structural constituents of ribosome and poly(A) RNA binding were enriched in the MRP family network. But MRPs work further than that. It has been reported that MRPs constitute oxidative phosphorylation (OXPHOS) enzyme complexes, leading to reactive oxygen species (ROS) overproduction^39^. They can be stimulated by the mTOR signaling pathway, as well as inactivation of the TCA cycle enzyme and AMP-activated kinase (AMPK) activities^40^. The upregulation of MRPs in malignancy could probably be explained by the “Warburg effect”, a phenomenon that cancer cells adapt to hypoxia and display high rates of glycolysis^41^. Through enhanced glycolytic ATP supply, PTEN inactivation, and activated protein kinase B activity^42^, MRP can contribute to cancerous metabolic alterations. On the contrary, some MRPs can retard cell cycle progression and induce apoptosis by regulation of p21WAF1/CIP1, p27Kip1, and p53^16,43,44^. The impact and interplay of our MRP signatures deserve further investigation.

In terms of prognostic values, MRPs have been identified as effective predictive markers for metastasis and recurrence^45^. However, most of the previous studies were characterized by a low number of MRPs and BC samples, or by insufficient follow-up data. For the first time, our study covered nearly all MRP family members in Cox proportional hazards regression. 7 MRP genes were identified as potential biomarkers for the prognostic evaluation of BC. Upregulated MRPL1, MRPL13, MRPS6, MRPS18C, and MRPS35 brought unfavorable survival outcomes for patients with BC. On the contrary, low levels of MRPL16 and MRPL40 implied a worse prognosis. Later, the most optimal combination was selected to construct an MRP-based prognostic model for BC survival prediction. In the nomogram, low MRPL16 and MRPL40 expression, as well as high MRPS18C and MRPS35 expression were acquired with more risk scores. MRPL40 has been proven to be elevated in BC^12^, but their prognostic effects remain unknown. These 4 MRP genes have seldom been studied and could serve as potential therapeutic candidates for treating BC in the future.

Our study comprehensively analyzed the expression, genomic alteration, and prognostic values of the MRP family in BC. MRPL16, MRPL40, MRPS18C, and MRPS35 were integrated into a novel prognostic nomogram to accurately predict the OS in BC patients. However, there are still some limitations. Although the MRP-based nomogram indicated good accuracy and conformity, external validation was required to confirm our results. Additionally, the association between the MRP gene family and drug therapeutic effect has not been studied. Further experiments are necessary to investigate the molecular mechanism of MRPs, especially MRPL16, MRPL40, MRPS18C, and MRPS35.

## Conclusion

MRP genes were primarily upregulated in BC, and MRP amplification was the most frequent alteration. They were associated with protein interactions in mitochondrial translational termination, elongation, and translation. MRPL16 and MRPL40 were positively associated with survival outcomes, while MRPS18C and MRPS35 were inversely correlated with OS. Besides thorough expression and prognosis analysis, an MRP-based nomogram was constructed. It yielded good discrimination and calibration, and therefore could accurately predict the survival outcomes for BC patients.

## Supplementary Information

Supplementary Figure 1.

Supplementary Figure 2.

Supplementary Figure 3.

Supplementary Table 1.

Supplementary Legends.

## Data Availability

All data generated or analyzed during this study are included in this published article and its supplementary information files.

## References

[1] Siegel RL, Miller KD, Fuchs HE, Jemal A (2021). Cancer statistics, 2021. CA Cancer J. Clin..

[2] Cheang MC (2009). Ki67 index, HER2 status, and prognosis of patients with luminal B breast cancer. J. Natl. Cancer Inst..

[3] Leone J, Leone J, Zwenger A, Vallejo C, Leone B (2019). Prognostic significance of tumor subtypes in women with breast cancer according to stage: A population-based study. Am. J. Clin. Oncol..

[4] Gómez-Acebo I (2020). Tumour characteristics and survivorship in a cohort of breast cancer: The MCC-Spain study. Breast Cancer Res. Treat..

[5] Blows FM (2010). Subtyping of breast cancer by immunohistochemistry to investigate a relationship between subtype and short and long term survival: A collaborative analysis of data for 10,159 cases from 12 studies. PLoS Med..

[6] Thewes B, Prins J, Friedlander M (2016). 70-Gene signature in early-stage breast cancer. N. Engl. J. Med..

[7] Pereira B (2016). The somatic mutation profiles of 2,433 breast cancers refines their genomic and transcriptomic landscapes. Nat. Commun..

[8] Paik S (2004). A multigene assay to predict recurrence of tamoxifen-treated, node-negative breast cancer. N. Engl. J. Med..

[9] Hanahan D, Weinberg RA (2011). Hallmarks of cancer: The next generation. Cell.

[10] Porporato PE, Filigheddu N, Pedro JMB, Kroemer G, Galluzzi L (2018). Mitochondrial metabolism and cancer. Cell Res..

[11] Mai N, Chrzanowska-Lightowlers ZM, Lightowlers RN (2017). The process of mammalian mitochondrial protein synthesis. Cell Tissue Res..

[12] Sotgia F (2014). Mitochondria “fuel” breast cancer metabolism: Fifteen markers of mitochondrial biogenesis label epithelial cancer cells, but are excluded from adjacent stromal cells. Cell Cycle.

[13] Liu Y (2021). Identification of a three-RNA binding proteins (RBPs) signature predicting prognosis for breast cancer. Front. Oncol..

[14] Li X (2021). HIF-1-induced mitochondrial ribosome protein L52: A mechanism for breast cancer cellular adaptation and metastatic initiation in response to hypoxia. Theranostics.

[15] Charafe-Jauffret E (2009). Breast cancer cell lines contain functional cancer stem cells with metastatic capacity and a distinct molecular signature. Cancer Res..

[16] Kim T (2013). Nuclear-encoded mitochondrial MTO1 and MRPL41 are regulated in an opposite epigenetic mode based on estrogen receptor status in breast cancer. BMC Cancer.

[17] Tang Z, Kang B, Li C, Chen T, Zhang Z (2019). GEPIA2: An enhanced web server for large-scale expression profiling and interactive analysis. Nucl. Acids Res..

[18] Barretina J (2012). The cancer cell line encyclopedia enables predictive modelling of anticancer drug sensitivity. Nature.

[19] Chandrashekar D (2017). UALCAN: A portal for facilitating tumor subgroup gene expression and survival analyses. Neoplasia (New York N. Y.).

[20] Chen F, Chandrashekar D, Varambally S, Creighton C (2019). Pan-cancer molecular subtypes revealed by mass-spectrometry-based proteomic characterization of more than 500 human cancers. Nat. Commun..

[21] Cerami E (2012). The cBio cancer genomics portal: An open platform for exploring multidimensional cancer genomics data. Cancer Discov..

[22] Modhukur V (2018). MethSurv: A web tool to perform multivariable survival analysis using DNA methylation data. Epigenomics.

[23] The Gene Ontology, C (2019). The gene ontology resource: 20 years and still GOing strong. Nucl. Acids Res..

[24] da Huang W, Sherman BT, Lempicki RA (2009). Bioinformatics enrichment tools: Paths toward the comprehensive functional analysis of large gene lists. Nucl. Acids Res..

[25] da Huang W, Sherman BT, Lempicki RA (2009). Systematic and integrative analysis of large gene lists using DAVID bioinformatics resources. Nat. Protoc..

[26] Szklarczyk D (2021). The STRING database in 2021: Customizable protein–protein networks, and functional characterization of user-uploaded gene/measurement sets. Nucl. Acids Res..

[27] Shannon P (2003). Cytoscape: A software environment for integrated models of biomolecular interaction networks. Genome Res..

[28] Goldman M (2020). Visualizing and interpreting cancer genomics data via the Xena platform. Nat. Biotechnol..

[29] Vyas S, Zaganjor E, Haigis MC (2016). Mitochondria and cancer. Cell.

[30] Yang Y (2016). Mitochondria and mitochondrial ROS in cancer: Novel targets for anticancer therapy. J. Cell. Physiol..

[31] Tao Z (2020). MRPL13 is a prognostic cancer biomarker and correlates with immune infiltrates in breast cancer. Onco Targets Ther..

[32] Xu YH (2020). Identification of candidate genes associated with breast cancer prognosis. DNA Cell Biol..

[33] Gao Y (2017). Down-regulation of MRPS23 inhibits rat breast cancer proliferation and metastasis. Oncotarget.

[34] Oviya RP (2021). Mitochondrial ribosomal small subunit proteins (MRPS) MRPS6 and MRPS23 show dysregulation in breast cancer affecting tumorigenic cellular processes. Gene.

[35] Cai M, Li H, Chen R, Zhou X (2021). MRPL13 promotes tumor cell proliferation, migration and EMT process in breast cancer through the PI3K-AKT-mTOR pathway. Cancer Manag. Res..

[36] Lee YK (2017). Lactate-mediated mitoribosomal defects impair mitochondrial oxidative phosphorylation and promote hepatoma cell invasiveness. J. Biol. Chem..

[37] Sartor CI, Dziubinski ML, Yu CL, Jove R, Ethier SP (1997). Role of epidermal growth factor receptor and STAT-3 activation in autonomous proliferation of SUM-102PT human breast cancer cells. Cancer Res..

[38] Liu L (2021). Arginine and lysine methylation of MRPS23 promotes breast cancer metastasis through regulating OXPHOS. Oncogene.

[39] Liu L (2018). MRPL33 and its splicing regulator hnRNPK are required for mitochondria function and implicated in tumor progression. Oncogene.

[40] Tong WH (2011). The glycolytic shift in fumarate–hydratase-deficient kidney cancer lowers AMPK levels, increases anabolic propensities and lowers cellular iron levels. Cancer Cell.

[41] Wang X, Peralta S, Moraes CT (2013). Mitochondrial alterations during carcinogenesis: A review of metabolic transformation and targets for anticancer treatments. Adv. Cancer Res..

[42] Piao L (2009). Association of LETM1 and MRPL36 contributes to the regulation of mitochondrial ATP production and necrotic cell death. Cancer Res..

[43] Yoo YA (2005). Mitochondrial ribosomal protein L41 suppresses cell growth in association with p53 and p27Kip1. Mol. Cell. Biol..

[44] Chen YC, Chang MY, Shiau AL, Yo YT, Wu CL (2007). Mitochondrial ribosomal protein S36 delays cell cycle progression in association with p53 modification and p21(WAF1/CIP1) expression. J. Cell. Biochem..

[45] Ózsvári B, Sotgia F, Lisanti MP (2020). First-in-class candidate therapeutics that target mitochondria and effectively prevent cancer cell metastasis: Mitoriboscins and TPP compounds. Aging (Albany N. Y.).

