# Supplementary figures and images for: Expression and prognosis analysis of mitochondrial ribosomal protein family in breast cancer
Source: Sci Rep. 2022 Jun 23;12:10658. doi: 10.1038/s41598-022-14724-7 (PMC9226049; doi:10.1038/s41598-022-14724-7)

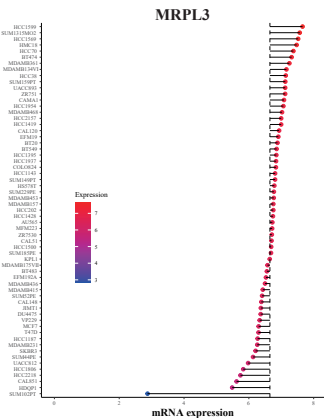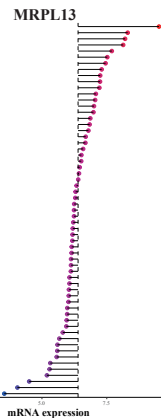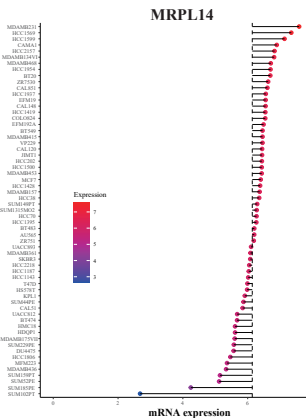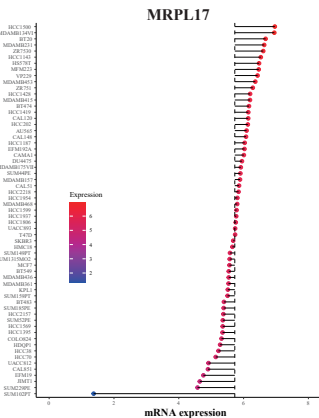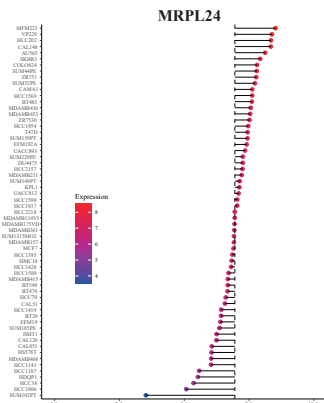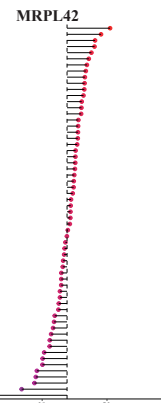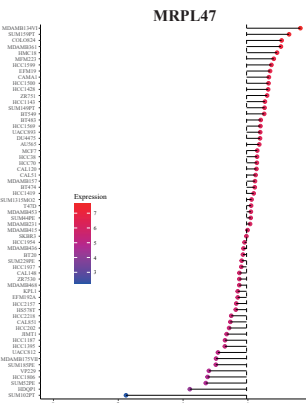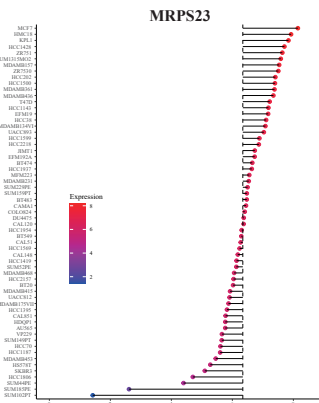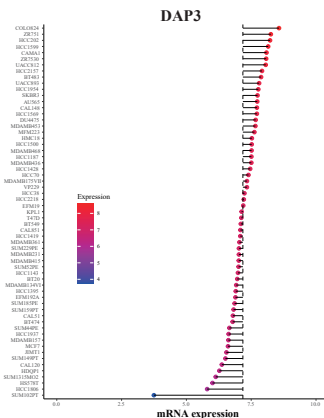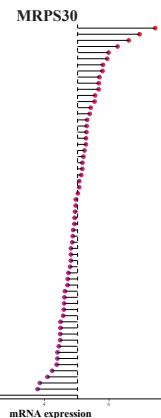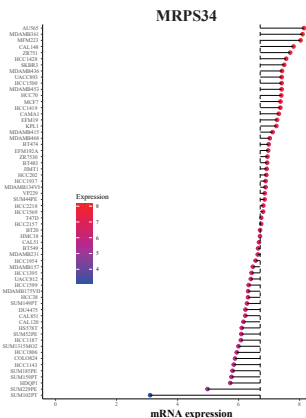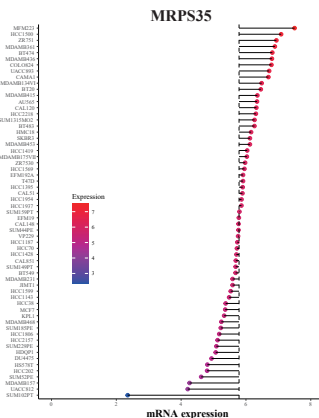

Supplement: Supplementary file 2 — Supplementary Figure 2. [file 41598_2022_14724_MOESM2_ESM.pdf]

MRPL3

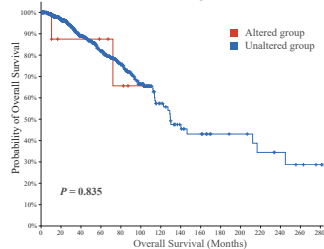

MRPL13

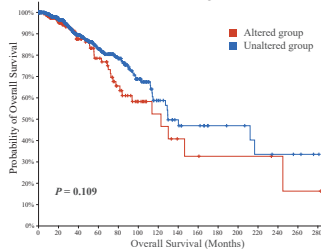

MRPL14

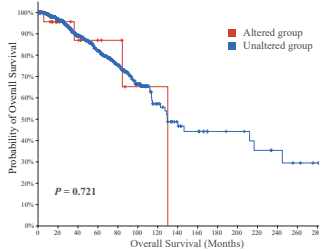

MRPL17

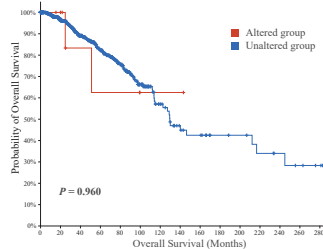

MRPL24

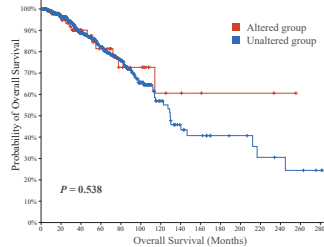

MRPL42

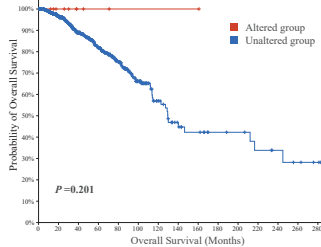

MRPL47

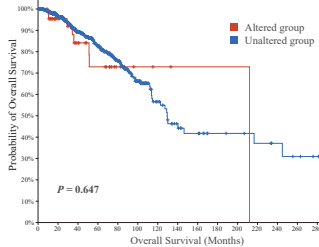

MRPS23

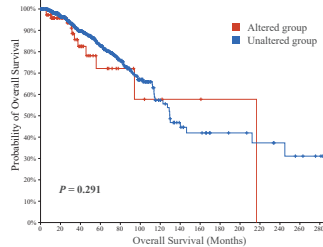

DAP3

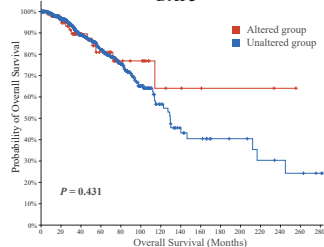

MRPS30

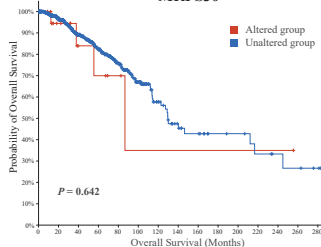

MRPS34

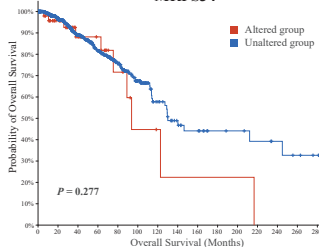

MRPS35

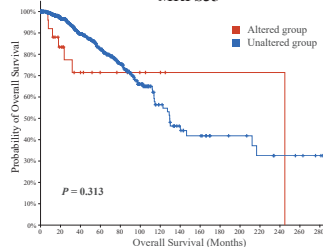

Supplement: Supplementary file 3 — Supplementary Figure 3. [file 41598_2022_14724_MOESM3_ESM.pdf]
