# Supplementary material for: Expression and prognosis analysis of mitochondrial ribosomal protein family in breast cancer
Source: Sci Rep. 2022 Jun 23;12:10658. doi: 10.1038/s41598-022-14724-7 (PMC9226049; doi:10.1038/s41598-022-14724-7)
Supplement: Supplementary file 5 — Supplementary Legends. [file 41598_2022_14724_MOESM5_ESM.docx]

**Supplementary Figure 1.** MRP gene expression across 33 types of primary tumors (CCLE). Box-and-whisker plots demonstrated the distribution of mRNA expression in a variety of cancer types, ordered by the corresponding median expression level (solid line=median level, box=inter-quartile range, error bar=mean standard deviation). The dotted line indicated the overall mean expression level.

**Supplementary Figure 2.** Expression of MRP genes in 61 breast cancer cell lines (CCLE). The upper dots in red represented higher mRNA expression, whereas the bottom dots in blue represented lower expression.

**Supplementary Figure 3.** Kaplan–Meier survival plots for patients with and without MRP genetic alteration (cBioportal).

**Supplementary Table 1.** Clinical and MRP expression information of breast cancer patients in TCGA dataset.
