# Supplementary material for: Expression and prognosis analysis of mitochondrial ribosomal protein family in breast cancer
Source: Sci Rep. 2022 Jun 23;12:10658. doi: 10.1038/s41598-022-14724-7 (PMC9226049; doi:10.1038/s41598-022-14724-7)
Supplement: Supplementary file 1 — Supplementary Figure 1. [file 41598_2022_14724_MOESM1_ESM.pdf]

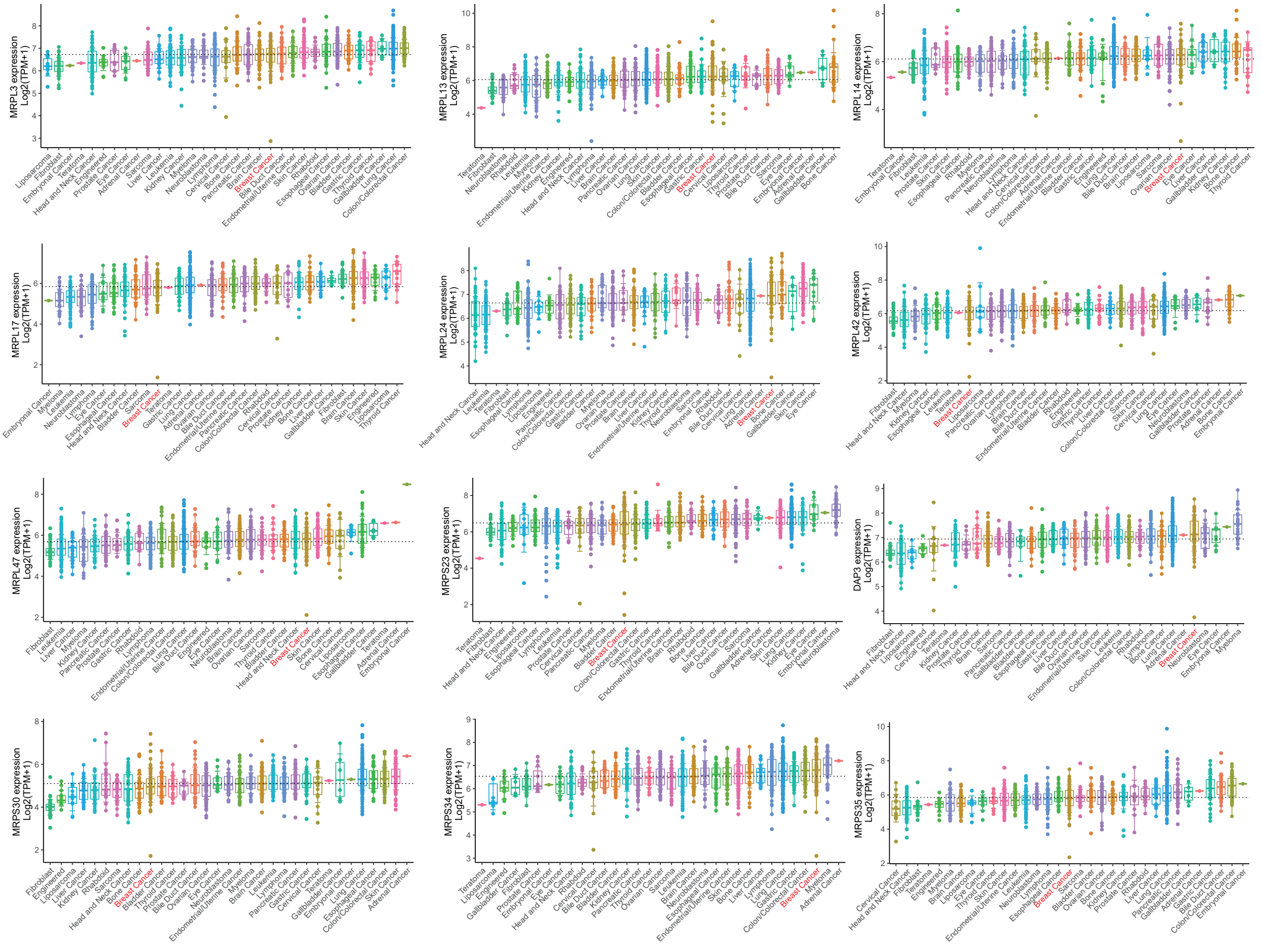
MRPL3 expression Log2(TPM+1)
Liposarcoma, Fibroblast, Embryonal Cancer, Teratoma, Head and Neck Cancer, Engineered, Prostate Cancer, Eye Cancer, Adrenal Cancer, Sarcoma, Liver Cancer, Leukemia, Kidney Cancer, Myeloma, Neuroblastoma, Lymphoma, Cervical Cancer, Bone Cancer, Pancreatic Cancer, Brain Cancer, Breast Cancer, Bile Duct Cancer, Endometrial/Uterine Cancer, Skin Cancer, Rhabdoid, Esophageal Cancer, Ovarian Cancer, Bladder Cancer, Gastric Cancer, Thyroid Cancer, Gallbladder Cancer, Lung Cancer, Colon/Colorectal Cancer
MRPL13 expression Log2(TPM+1)
Teratoma, Fibroblast, Neuroblastoma, Rhabdoid, Leukemia, Myeloma, Endometrial/Uterine Cancer, Kidney Cancer, Engineered, Head and Neck Cancer, Lymphoma, Liver Cancer, Brain Cancer, Pancreatic Cancer, Ovarian Cancer, Lung Cancer, Colon/Colorectal Cancer, Skin Cancer, Bladder Cancer, Esophageal Cancer, Gastric Cancer, Breast Cancer, Cervical Cancer, Liposarcoma, Thyroid Cancer, Prostate Cancer, Bile Duct Cancer, Sarcoma, Eye Cancer, Embryonal Cancer, Adrenal Cancer, Gallbladder Cancer, Bone Cancer
MRPL14 expression Log2(TPM+1)
Teratoma, Embryonal Cancer, Fibroblast, Leukemia, Prostate Cancer, Skin Cancer, Esophageal Cancer, Rhabdoid, Myeloma, Pancreatic Cancer, Neuroblastoma, Lymphoma, Head and Neck Cancer, Cervical Cancer, Colon/Colorectal Cancer, Adrenal Cancer, Endometrial/Uterine Cancer, Bladder Cancer, Gastric Cancer, Engineered, Lung Cancer, Bile Duct Cancer, Brain Cancer, Liposarcoma, Sarcoma, Ovarian Cancer, Breast Cancer, Eye Cancer, Liver Cancer, Gallbladder Cancer, Kidney Cancer, Bone Cancer, Thyroid Cancer
MRPL17 expression Log2(TPM+1)
Embryonal Cancer, Myeloma, Leukemia, Neuroblastoma, Lymphoma, Eye Cancer, Esophageal Cancer, Head and Neck Cancer, Bladder Cancer, Sarcoma, Breast Cancer, Teratoma, Gastric Cancer, Lung Cancer, Adrenal Cancer, Ovarian Cancer, Bile Duct Cancer, Endometrial/Uterine Cancer, Pancreatic Cancer, Colon/Colorectal Cancer, Rhabdoid, Cervical Cancer, Prostate Cancer, Kidney Cancer, Bone Cancer, Liver Cancer, Gallbladder Cancer, Fibroblast, Brain Cancer, Skin Cancer, Engineered, Liposarcoma, Thyroid Cancer
MRPL24 expression Log2(TPM+1)
Head and Neck Cancer, Leukemia, Teratoma, Fibroblast, Esophageal Cancer, Lymphoma, Liposarcoma, Engineered, Pancreatic Cancer, Colon/Colorectal Cancer, Gastric Cancer, Bladder Cancer, Myeloma, Ovarian Cancer, Prostate Cancer, Endometrial/Uterine Cancer, Brain Cancer, Liver Cancer, Kidney Cancer, Thyroid Cancer, Neuroblastoma, Sarcoma, Embryonal Cancer, Rhabdoid, Bile Duct Cancer, Cervical Cancer, Lung Cancer, Adrenal Cancer, Breast Cancer, Bone Cancer, Gallbladder Cancer, Skin Cancer, Eye Cancer
MRPL42 expression Log2(TPM+1)
Fibroblast, Head and Neck Cancer, Myeloma, Kidney Cancer, Esophageal Cancer, Leukemia, Teratoma, Breast Cancer, Liposarcoma, Pancreatic Cancer, Ovarian Cancer, Lymphoma, Brain Cancer, Bile Duct Cancer, Endometrial/Uterine Cancer, Bladder Cancer, Rhabdoid, Engineered, Gastric Cancer, Thyroid Cancer, Colon/Colorectal Cancer, Liver Cancer, Sarcoma, Skin Cancer, Cervical Cancer, Lung Cancer, Eye Cancer, Neuroblastoma, Gallbladder Cancer, Prostate Cancer, Adrenal Cancer, Bone Cancer, Embryonal Cancer
MRPL47 expression Log2(TPM+1)
Fibroblast, Leukemia, Liver Cancer, Myeloma, Kidney Cancer, Pancreatic Cancer, Prostate Cancer, Gastric Cancer, Rhabdoid, Lymphoma, Endometrial/Uterine Cancer, Colon/Colorectal Cancer, Lung Cancer, Bile Duct Cancer, Engineered, Eye Cancer, Neuroblastoma, Brain Cancer, Ovarian Cancer, Sarcoma, Thyroid Cancer, Bladder Cancer, Head and Neck Cancer, Breast Cancer, Skin Cancer, Bone Cancer, Cervical Cancer, Liposarcoma, Esophageal Cancer, Gallbladder Cancer, Teratoma, Adrenal Cancer, Embryonal Cancer
MRPS23 expression Log2(TPM+1)
Teratoma, Fibroblast, Head and Neck Cancer, Engineered, Liposarcoma, Esophageal Cancer, Lymphoma, Leukemia, Prostate Cancer, Cervical Cancer, Pancreatic Cancer, Myeloma, Bladder Cancer, Breast Cancer, Colon/Colorectal Cancer, Gastric Cancer, Thyroid Cancer, Endometrial/Uterine Cancer, Brain Cancer, Rhabdoid, Bone Cancer, Liver Cancer, Bile Duct Cancer, Ovarian Cancer, Sarcoma, Gallbladder Cancer, Adrenal Cancer, Skin Cancer, Lung Cancer, Kidney Cancer, Eye Cancer, Embryonal Cancer, Neuroblastoma
DAP3 expression Log2(TPM+1)
Fibroblast, Head and Neck Cancer, Liposarcoma, Engineered, Cervical Cancer, Teratoma, Kidney Cancer, Prostate Cancer, Thyroid Cancer, Brain Cancer, Sarcoma, Pancreatic Cancer, Gallbladder Cancer, Esophageal Cancer, Bladder Cancer, Gastric Cancer, Liver Cancer, Bile Duct Cancer, Endometrial/Uterine Cancer, Ovarian Cancer, Skin Cancer, Colon/Colorectal Cancer, Leukemia, Rhabdoid, Lymphoma, Bone Cancer, Lung Cancer, Adrenal Cancer, Breast Cancer, Neuroblastoma, Eye Cancer, Embryonal Cancer, Myeloma
MRPS30 expression Log2(TPM+1)
Fibroblast, Engineered, Liposarcoma, Liver Cancer, Kidney Cancer, Rhabdoid, Head and Neck Cancer, Sarcoma, Breast Cancer, Bone Cancer, Bladder Cancer, Thyroid Cancer, Prostate Cancer, Bile Duct Cancer, Ovarian Cancer, Eye Cancer, Endometrial/Uterine Cancer, Neuroblastoma, Brain Cancer, Myeloma, Leukemia, Pancreatic Cancer, Lymphoma, Gastric Cancer, Cervical Cancer, Teratoma, Gallbladder Cancer, Embryonal Cancer, Esophageal Cancer, Colon/Colorectal Cancer, Lung Cancer, Skin Cancer, Adrenal Cancer
MRPS34 expression Log2(TPM+1)
Teratoma, Liposarcoma, Engineered, Gallbladder Cancer, Fibroblast, Prostate Cancer, Embryonal Cancer, Eye Cancer, Head and Neck Cancer, Rhabdoid, Cervical Cancer, Bile Duct Cancer, Bladder Cancer, Kidney Cancer, Pancreatic Cancer, Thyroid Cancer, Ovarian Cancer, Sarcoma, Leukemia, Brain Cancer, Neuroblastoma, Esophageal Cancer, Endometrial/Uterine Cancer, Skin Cancer, Bone Cancer, Liver Cancer, Lymphoma, Lung Cancer, Gastric Cancer, Colon/Colorectal Cancer, Breast Cancer, Myeloma, Adrenal Cancer
MRPS35 expression Log2(TPM+1)
Cervical Cancer, Head and Neck Cancer, Fibroblast, Teratoma, Engineered, Myeloma, Brain Cancer, Liposarcoma, Eye Cancer, Thyroid Cancer, Skin Cancer, Endometrial/Uterine Cancer, Leukemia, Neuroblastoma, Lymphoma, Esophageal Cancer, Breast Cancer, Sarcoma, Bladder Cancer, Ovarian Cancer, Bone Cancer, Kidney Cancer, Prostate Cancer, Rhabdoid, Liver Cancer, Lung Cancer, Pancreatic Cancer, Gallbladder Cancer, Adrenal Cancer, Gastric Cancer, Bile Duct Cancer, Colon/Colorectal Cancer, Embryonal Cancer
